# Glutathione peroxidases as oncotargets

**DOI:** 10.18632/oncotarget.20278

**Published:** 2017-08-16

**Authors:** Yang Jiao, Yirong Wang, Shanchun Guo, Guangdi Wang

**Affiliations:** ^1^ Department of Stomatology, PLA Army General Hospital, Beijing, P.R. China; ^2^ State Key Laboratory of Military Stomatology & National Clinical Research Center for Oral Diseases & Shaanxi Key Laboratory of Oral Diseases, Department of Operative Dentistry and Endodontics, School of Stomatology, The Fourth Military Medical University, Xi’an, P.R. China; ^3^ RCMI Cancer Research Center and Department of Chemistry, Xavier University of Louisiana, New Orleans, LA, USA

**Keywords:** glutathione peroxidases, oxidative stress, reactive oxygen species, carcinogenesis, drug target

## Abstract

Oxidative stress is a disturbance in the equilibrium among free radicals, reactive oxygen species, and endogenous antioxidant defense mechanisms. Oxidative stress is a result of imbalance between the production of reactive oxygen and the biological system’s ability to detoxify the reactive intermediates or to repair the resulting damage. Mounting evidence has implicated oxidative stress in various physiological and pathological processes, including DNA damage, proliferation, cell adhesion, and survival of cancer cells. Glutathione peroxidases (GPxs) (EC 1.11.1.9) are an enzyme family with peroxidase activity whose main biological roles are to protect organisms from oxidative damage by reducing lipid hydroperoxides as well as free hydrogen peroxide. Currently, 8 sub-members of GPxs have been identified in humans, all capable of reducing H_2_O_2_ and soluble fatty acid hydroperoxides. A large number of publications has demonstrated that GPxs have significant roles in different stages of carcinogenesis. In this review, we will update recent progress in the study of the roles of GPxs in cancer. Better mechanistic understanding of GPxs will potentially contribute to the development and advancement of improved cancer treatment models.

## INTRODUCTION

Oxidative stress is essentially an imbalance among reactive oxygen species (ROS), free radicals (FR), and endogenous antioxidant defense mechanisms in the cell. Cellular molecules and components will ultimately lose cells viability by severe oxidative damage [1-2].

Oxidative stress is involved in various physiological processes, including cell adhesion, proliferation, DNA damage, and survival. Oxidative stress is also involved in a large number of pathological states, such as Alzheimer’s disease [3-5], Parkinson’s disease [6-7], atherosclerosis [8-9], heart failure [10-11], fragile X syndrome [12], myocardial infarction [13], Sickle cell disease [14], hepatic encephalopathy [15-16], as well as carcinogenesis [17-20]. Glutathione peroxidases (GPxs) is an enzyme family which has the ability to reduce free hydrogen peroxide to water and reduce lipid hydroperoxides to their corresponding alcohols and its main biological roles are to protect organisms from oxidative stress damage [21-24]. GPxs include five members and have been found in different tissues of the body and cell fractions. As expected, GPxs, a major defender against *oxidative stress* were also reported to be involved in Parkinson’s disease [25-27], Alzheimer’s disease [28-30], atherosclerosis [31-32], myocardial infarction [33-34], heart failure [35-36], Sickle cell disease [37], as well as carcinogenesis [24, 38-39]. In this review we analyzed the role and function of GPxs in mammalian cells, in the regulation of stem cells and cancer stem cells (CSC), discussed the GPxs-mediated signaling pathways and their potential as biomarkers and drug targets. Additionally, we discussed molecular mechanisms of GPxs in CSC, carcinogenesis, as well as its crosstalk with other signaling pathways.

## EXPRESSION AND FUNCTION OF GPXS IN MAMMALIAN CELLS

GPxs in vertebrates are comprised of 8 sub-members, i.e. GPx 1-8. The molecular mass of the active purified mammalian GPx1, a tetramer of identical subunits of ∼22-23 kDa, has been estimated to be between 83 and 95 kDa [40-42]. GPx1 is ubiquitously expressed and predominantly found in the cytosol [43] and mitochondria [44]. Recently, exosome-derived GPx1 was also found to be required for the recovery of hepatic oxidant injury [45]. Different locations of the GPx1 may correspond to different functions, i.e. GPx1 in cytosol may scavenge hydrophilic peroxide species such as H_2_O_2_ [43], whereas in mitochondria it may protect mitochondrial DNA from oxidative damage [44, 46]. GPx1 is considered as a major antioxidant enzyme within the GPx family, although GPx1-/- mice apparently were healthy, fertile and showed no increased sensitivity to hyperoxia and could compensate for mild oxidative stress [47].

GPx2 is detected in the gastrointestinal system of mammals and also expressed in human livers [48-49]. Its expression pattern suggests that major function of GPx2 is against ROS derived from the gut. GPx2 knockout mice do not develop an aberrant phenotype before birth, but GPx2 gene is able to make up for the lack of GPx1 gene expression in the ileum epithelium [50] .

GPx3, mainly expressed in the proximal tubuli of the kidney, is a secreted plasma protein and was found in most extracellular fluids [43, 51]. GPx3-/- mice show no abnormal phenotype throughout their life times, and GPx3 is not involved in selenium metabolism [52]. However, GPx3 may have glutathione peroxidase activity in the cortical peritubular space, since the specific binding of a large pool of GPx3 is seen in the basement membranes in the kidney cortex [52].

Three distinct GPx4 isoforms with different subcellular localizations are detected in mouse and rat: mitochondrial GPx4 (mGPx4), cytosolic GPx4, and nuclear GPx4 (nGPx4) [53]. Cytosolic GPx4 is implicated for cell survival and embryonic development, while nGPx4 and mGPx4 have been essential in male fertility and spermatogenesis [53]. The key features of GPx4 function are its dual anti-oxidative and anti-apoptotic activities [54]. In developing embryos GPx4 expression correlates with areas of reduced apoptosis in developing limbs [55]. In contrast to GPx1-3, all GPx4 knockout strategies fail to reproduce viable homozygous offspring [56-57].

GPx5, the closest homologue to GPx3, is detected in the epididymis of reproductive tract in the mammalian male, and is androgen-regulated [58]. While the kinetics and substrate specificities of GPx5 are not fully understood, the function related to the maintenance of sperm DNA integrity [59]. Thus, GPx5 might be a potent antioxidant scavenger that protects spermatozoa from oxidative injuries that can potentially compromise their integrity and embryo viability [59].

GPx6, as a putative odorant-binding and metabolizing enzyme, was identified by in silico analysis [60]. Its detection appears to be restricted to the embryos and the Bowman’s glands [61]. Since GPx6 has not been purified, the knowledge on GPx6 is very limited.

GPx7 with a cysteine instead of Sec in the catalytic center is a cytoplasmic protein with molecular mass of approximately 22 kDa [62]. GPx7 has little glutathione peroxidase activity *in vitro* [62] and is detected in the lumen of the endoplasmic reticulum [63]. Recently, GPx7 was identified as a stress sensor that transmits oxidative stress signals and it is critical for releasing excessive ER stress by increasing GRP78 chaperone activity [64].

GPx8 as a novel member belonging to the GPx family, has been identified in a phylogenetic analysis in amphibia and mammalia [65]. GPx8 is a membrane protein, lung-abundant enzyme and is detected in endoplasmic reticulum [63, 66]. However, little is known about its role.

As described above, the functions of most of these proteins are not completely known, but they may all be capable of reducing hydroperoxides: ROOH + 2GSH→ROH + H_2_O + GSSG. Thus, a common function of GPxs should be related to the metabolism of hydroperoxides.

## GPXS IN THE REGULATION OF STEM CELLS

Stem cell (SC) research has obtained increasing attention in the past decade due to their invaluable clinical potentials to cure genetic disorders, degenerative diseases, and even cancers. SCs present in all multicellular organisms, can self-renew to produce more SCs and can divide and differentiate into diverse specialized cell types [67]. Although considerable studies have been performed in elucidating the molecular mechanisms in the regulation of self-renewal and differentiation of all kinds of SCs, relatively little effort has been made in investigating the metabolic aspects of SCs [67-68].

Since both embryonic SCs and adult SCs are sources of different types of mature cells, SCs must need specific protection from the long-term effects of oxidative damage and ROS. The intersection of SC function and ROS was proven from the study of mice that lack the function of the gene ataxia telangiectasia mutated (Atm). Most Atm knockout mice die at early, whereas a small percentage of mice survive [69]. Further analysis showed that Atm−/− hematopoietic SCs (HSC) had a severe defect in self-renewal and a marked increase in ROS levels [69]. Analysis of HSCs from conditional knockout of FoxO1, FoxO3, and FoxO4 mice showed an increase in ROS levels and a decline in long-term repopulating activity [70]. In a recent study, Prdm16 deficiency was proven to lead to changes in the levels of ROS, depletion of SCs, altered cell-cycle distribution in the haematopoietic and nervous systems and an increase in ROS levels [71]. Prdm16 is a transcription factor that Prdm16 binds to the Hgf promoter, and Hgf expression declined in the absence of Prdm16 [72-74]. In neural SCs, Prdm16 binds to the Hgf promoter, and Hgf expression declined in the absence of Prdm16 [71]. Thus, Prdm16, promotes SC maintenance and self-renewal in multiple tissues, partly by modulating oxidative stress. All these findings involving Atm, FoxO, as well as Prdm16 indicate that there is a strong correlation between the maintenance of SC function and ROS homeostasis.

In recent years, the role of GPxs in the regulation of SCs is finally being recognized. GPx1 is an important antioxidant enzyme in preventing the harmful accumulation of intracellular hydrogen peroxide. Although GPx1-/- mice were apparently healthy [47], GPx1 was still considered to be the most important member of GPx family in modulating redox-mediated responses and cellular oxidant stress, as well as in the regulation of SCs [21, 75]. Localization of GPx2 in the intestinal crypt epithelium points to a specific function of this particular GPx in the gastrointestinal SC regulation. Loss of GPx2 led to an increase in apoptotic cells at colonic crypt bases, an area critical for the self-renewal of the intestinal epithelium [76]. These results indicate a role for GPx2 in regulating intestinal mucosal SC homeostasis. Recently, GPx3 was demonstrated that it is essential for human skeletal muscle precursor cell survival [77]. In an early report, in normal HSCs without function of GPx3 were much less competitive *in vivo* than in control cells. While HSCs overexpressing GPx3 with overexpression of the self-renewal genes Prdm16 or Hoxb4 boosted GPx3 expression were significantly more competitive than control cells [78]. As mentioned above, GPx4 is essential for embryonic SC development andsurvival, especially in spermatogenesis and male fertility [53], and GPx5 may also have a role in the mammalian male SC regulation [58]. So far, there has been no report about the role of GPx6, 7 and 8 in SC regulation.

## GPXS IN SEVERAL TYPES OF TUMOR

Changes in GPx levels in several types of tumor have been reported. However, it remains unknown which of the GPx level changes are causative factors in caner progression. The role of GPx1 in tumor and its potential future therapies has been recently reviewed and discussed [21, 61]. GPx1 was reported to prevent oxidative DNA mutations, thus GPx1 may prevent tumorigenesis [79]. Overexpressed GPx1 reduced growth of tumors indicates that it has a role of the protective effect in tumorigenesis [80]. GPx1 polymorphism in a number of malignancy subjects showed that it may be an important factor modifying oxidative stress response [81-86]. The dual role of GPx2 in tumorigenesis has been reviewed recently [38, 61]. Overexpression of GPx2 was observed in several tumors including colorectal cancer [87-88], Barrett’s esophagus carcinoma [89-90], and lung cancer [91], indicating that GPx2 may be an oncogene. However, GPx2 was also observed to be down-regulated in prostatic intraepithelial neoplasia [91], indicating GPx2 may play a more complex role in tumorigenesis. GPx3 is considered to be a novel tumor suppressor, since hypermethylation of the GPx3 was detected in tumor samples from patients with Barrett’s esophagus [92-94], endometrial [95] and prostate cancer [96], and down-regulation was generally correlated with worse prognosis. Since changes in *GPx3* hypermethylation are reversible, drug treatment through demethylation may be a useful strategy to delay carcinogenesis and progression. In fact, the expression of GPX3 mRNA and protein was restored in several types of cancer cells after treatment with 5-aza-2’-deoxycytidine and [94, 97-98]. This method may bring new direction and potential for cancer treatment through gene-targeted therapy. GPx4 is also considered to be a tumor suppressor, since it was down-regulated in pancreatic [80] and breast cancer [99]. In addition, GPx4 overexpression reduced fibrosarcoma cell growth [100]. So far, there is no report on the role of GPx5, 6, 7 and 8 in tumorigenesis.

## GPXS IN CANCER STEM CELLS

Cancer cells are believed to originate from a small subpopulation of cells that have a high capacity of aberrantly self-renewal and differentiation, namely tumor-initiating cells or cancer stem cells (CSCs) [101-102]. CSCs have characteristics associated with normal stem cells, specifically the ability to cause the heterogeneous lineages or cell types [103-104]. Although their role and existence remain controversial, the reports of CSCs in mouse tumors also support this concept [105-106]. Unlike SCs, which have the differentiation process leading to specialized progenies with no proliferative potential, CSCs give rise to progenies that do not undergo terminal differentiation but instead exhibit uncontrolled proliferation. There are also differences in cell-cycle properties, mode of division, replicative potential, and DNA damage repairs. Through deregulation of the self-renewal process, CSCs initiate and drive carcinogenesis and contribute cellular heterogeneity [107]. Thus, CSCs may be a risk biomarker for carcinogenesis [108]. Previous studies also demonstrated that CSCs in solid tumors that are responsible for tumor initiation, progression, metastasis and drug resistance [109-110].

In comparison with cancer cells or SCs, relatively little is known about the ROS in CSCs. Similar to SCs, CSCs also contain lower intracellular ROS contents due to the increased production of free radical scavenging systems [111-112]. In addition, the unchecked ROS production may play a role in the leukemic initiation [113-114]. CSCs might have a high antioxidant capacity to keep cellular ROS at a moderate level. Breast CSCs exhibit an enhanced ROS defense system and lower levels of basal and radiation-induced ROS which may be associated with tumorigenicity and resistance to radiation [111]. Recently, CD13/Aminopeptidase N, a scavenger enzyme in the ROS metabolic pathway [115-116], was demonstrated to play a role in supporting the survival of CSCs and that there is an EMT-associated reduction in ROS elevation [117]. CSCs display an EMT phenotype and are resistant to current therapies. More recently, Dong et al showed that these phenotypes are stimulated by a metabolic switch to glucose metabolism, resulting in decreased ROS production in basal-like breast cancer [118].

Mitochondria and NADPH oxidases (NOX) are intracellular sources of H_2_O_2_ or other hydroperoxides (ROOH). A number of studies have established that H_2_O_2_ and/or ROOH are able to activate cyclooxygenase-2 (COX-2), one of the cyclooxygenases that catalyze a critical step in the formation of proinflammatory prostaglandins, e.g., PGE2 [24, 119-120]. COX-2 is over-expressed in cancer tissues [121-122], as well as in several kinds of CSCs [123-125]. PGE2, the main product of the COX-2 cascade play a role in the acute inflammatory response [126] and in tumor cell proliferation and invasion [127]. PGE2 enhances tumor cell proliferation and inhibits apoptosis by the activation of pro-survival pathways such as the PI3K/Akt or the Ras-MAPK/ERK pathways. PGE2 also supports cancer cell migration, invasion [126-127], and angiogenesis [128-129]. Moreover, PGE2 induces CSCs through the Wnt pathway [130-131].

**Figure 1:**
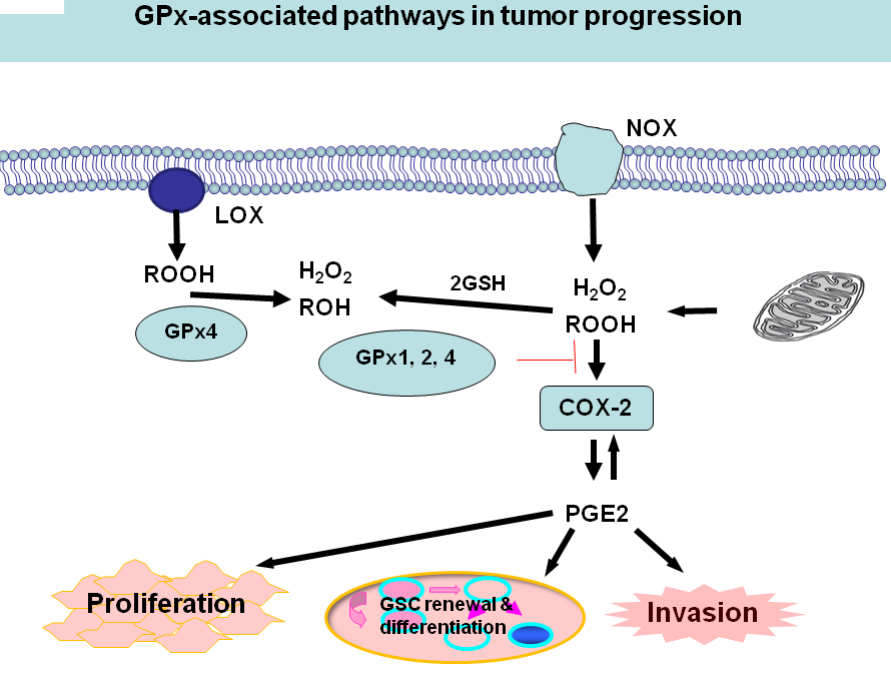
GPxs and COX/LOX activity Mitochondria and NADPH oxidases (NOX) are intracellular sources of H_2_O_2_ or other hydroperoxides (ROOH). H_2_O_2_ and ROOH are reduced by all GPx1, 2, and 4 and also by GPx3. Hydroperoxides activate COX-2, which in principle, is inhibited by all GPxs, preferentially; however, by GPx4. COX-2 forms PGE2, which in an autocrine loop can induce the expression of COX-2 to further increase PGE2 production to promote cancer cell proliferation, invasion, self-renewal, and differentiation of CSCs.

An imbalance or dysregulation of ROS levels may generate cells with abnormal growth, therefore potentially tumorigenic. Thus, a precise balance between processes generating ROS and those decomposing ROS is critical for tumor development including CSC self-renewal and differentiation. One tier of the cellular protective system against ROS constitutes the GPx family. Due to the significant role GPxs play in modulating redox-mediated responses and cellular oxidant stress, certain members of GPx may also play a critical role in CSC self-renewal and differentiation [61, 132-133]. Glioma stem cell lines expressing active GPx1 might decrease ROS level in Glioma CSCs and resist ROS/RNS-mediated cell death, thus creates a carcinoma stem cell niche [134]. Herault et al also reported that the expression of ROS scavenger, GPx3 associates with the frequency of leukemia stem cells (LSCs) in induced leukemias [78]. In addition, GPxs can potentially directly decrease H_2_O_2_ and/or ROOH level, then inactivate COX-2 and PEG2 and finally modulate CSCs (Figure 1). Recently, the Weinberg’s group showed thattumor cells strongly induced the COX-2/microsomal prostaglandin-E synthase-1 (mPGES-1)/PGE2 axis in MSCs [135]. GPxs, such as GPx1 also significantly impact human endothelial cell activation and proinflammatory cytokine-induced redox signaling [136-138], while endothelial cell activation is considered to be able to create a niche that helps promote self-renewal of CSCs [139-140]. Therefore, GPxs can potentially modulate CSCs through multiple pathways.

## CONCLUSIONS

A lot of knowledge has been learned about the GPx family in redox biology and cancer biology since their discoveries as crucial antioxidant enzymes that inactivate peroxides. GPxs participate in balancing the H_2_O_2_ homeostasis in signaling cascades and in tumorigenesis. GPx1, GPx2, GPx3 and 4 are also implicated in self-renewal and differentiation of stem cells and CSCs through multiple pathways. The current challenge is to unravel how the different GPx members can exert highly specific biological functions. The functions of some members of GPx, such as GPx6, 7 and 8 are still not known and require further research.
